# Robust prediction of irritability from brain functional connectivity in a transdiagnostic psychiatric sample of children and adolescents

**DOI:** 10.1192/j.eurpsy.2026.10253

**Published:** 2026-07-31

**Authors:** X. Liu, Y. Zhang, J. Chen, K. Wang, Y. Yang, X. Zhu, W. Zhang

**Affiliations:** 1 School of Psychology; 2 Shandong Provincial Key Laboratory of Brain Science and Mental Health, Shandong Normal University; 3 Shandong Mental Health Center, Shandong University; 4 Medical Science and Technology Innovation Center, Shandong First Medical University & Shandong Academy of Medical Sciences, Jinan, China

## Abstract

**Introduction:**

Irritability is a common and impairing transdiagnostic symptom across multiple psychiatric disorders in children and adolescents, including ADHD, generalized anxiety disorder, and depression. It is often manifested as a stable, trait-like phenotype that significantly impacts daily functioning and long-term outcomes. Despite its clinical relevance, the underlying neural mechanisms—particularly those that generalize across diagnostic categories—remain poorly understood.

**Objectives:**

This study aimed to identify transdiagnostic neural markers of irritability in a large developmental sample using resting-state functional connectivity. Specifically, we sought to determine whether functional network connectivity patterns could predict irritability severity and to validate their generalizability across both internal subsamples and an external clinical cohort.

**Methods:**

We analyzed resting-state fMRI data from 1143 children and adolescents (age = 11.65 ± 3.47 years) from the Healthy Brain Network project, encompassing diagnoses such as ADHD, depression, anxiety, and autism spectrum disorder. Irritability was measured using the Affective Reactivity Index. Connectome-based predictive modeling (CPM) was employed to identify functional networks associated with irritability. Internal validation was conducted using two random subsamples and an alternative brain atlas. Furthermore, an support vector machine (SVM) classifier was applied to an independent depression cohort (n = 129) to externally validate the robustness of the identified networks.

**Results:**

The positive predictive network for irritability primarily featured connections between the fronto-parietal network and other networks, whereas the negative network involved connections between the basal ganglia network and other networks. Internal validations confirmed that both the fronto-parietal network and the basal ganglia network consistently predicted irritability. External validation in the depression cohort further supported the role of these networks in irritability, successfully differentiating between high- and low-anger groups using SVM classification.

**Conclusions:**

Our findings underscore the central roles of the fronto-parietal network—implicated in cognitive control—and the basal ganglia network—associated with motivational and emotional processes—in pediatric irritability across diagnostic boundaries. These networks may reflect a developmental imbalance between top-down regulation and bottom-up emotional responding, offering potential neural targets for early intervention and transdiagnostic treatment strategies.

**Disclosure of Interest:**

None Declared

